# The Application of Telemedicine in Orthopedic Surgery in Singapore: A Pilot Study on a Secure, Mobile Telehealth Application and Messaging Platform

**DOI:** 10.2196/mhealth.3303

**Published:** 2014-06-05

**Authors:** Zubin Jimmy Daruwalla, Keng Lin Wong, Joseph Thambiah

**Affiliations:** ^1^National University Hospital, SingaporeDepartment of Orthopaedic SurgeryNational University of SingaporeSingaporeSingapore

**Keywords:** MyDoc, personal data protection, secure messaging, telehealth, telemedicine

## Abstract

**Background:**

The application of telemedicine has been described for its use in medical training and education, management of stroke patients, urologic surgeries, pediatric laparoscopic surgeries, clinical outreach, and the field of orthopedics. However, the usefulness of a secure, mobile telehealth application, and messaging platform has not been well described.

**Objective:**

A pilot study was conducted to implement a health insurance portability and accountability act (HIPAA) compliant form of communication between doctors in an orthopedic clinical setting and determine their reactions to MyDoc, a secure, mobile telehealth application, and messaging platform.

**Methods:**

By replacing current methods of communication through various mobile applications and text messaging services with MyDoc over a six week period, we gained feedback and determined user satisfaction with this innovative system from questionnaires handed to the program director, program coordinator, one trauma consultant, all orthopedic residents, and six non-orthopedic residents at the National University Hospital in Singapore.

**Results:**

Almost everyone who completed the questionnaire strongly agreed that MyDoc should replace current systems of peer to peer communication in the hospital. The majority also felt that the quality of images, videos, and sound were excellent. Almost everyone agreed that they could communicate easily with each other and would feel comfortable doing so routinely. The majority felt that virtual consults through MyDoc should be made available to inpatients as well as outpatients to potentially lessen clinic loads and provide a secure manner in which patients can communicate with their primary teams any time convenient to both. It was also agreed by most that the potential of telerounding had advantages, especially on weekends as a supplement to normal rounds.

**Conclusions:**

Potential uses of MyDoc in an orthopedic clinical setting include HIPAA-compliant peer to peer communication, clinical outreach in the setting of trauma, supervision in the operating room or watching procedures being performed remotely, providing both patient and parent reassurance in pediatric orthopedic patients, and finally in the setting of outpatient clinics. With our pilot study having excellent results in terms of acceptance and satisfaction, the integration of a secure, mobile telehealth application, and messaging platform, not only in the orthopedic department but also the hospital in general, has an exciting and limitless potential. More so in this era where downsizing hospital costs is beneficial, doing so may also be mandatory in order to comply with the soon to be introduced personal data protection act.

## Introduction

Recent advances in technology have challenged the ideology and the means by which traditional healthcare is provided. The application of telemedicine has been described for its use in medical training and education [1,2], management of stroke patients [3,4], urologic surgeries [5], pediatric laparoscopic surgeries [6], clinical outreach [5,7], and in the field of orthopedics [8]. However, its practicality in the field of secure messaging and other necessary functions in orthopedic surgery is yet to be described. Recently “MyDoc”, a private but government-incubated healthcare platform startup was introduced into the market. This platform integrated a number of functions, including a patient diary, virtual teleconsults through a live video conferencing system accessible from any location in the presence of an Internet or a Wi-Fi connection, and a secure communications application. The objective of our pilot study was to determine staff reaction to MyDoc and its secure, mobile telehealth application and alternative messaging platform at an orthopedic clinical setting in Singapore.

## Methods

**Figure 1:**
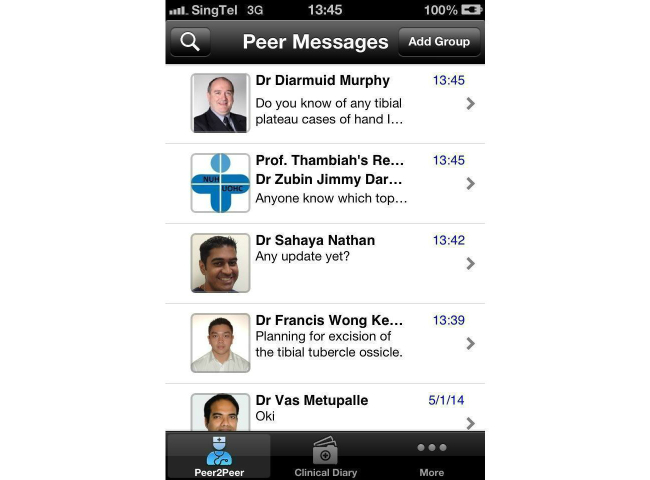
Screenshot of the personal messages user interface.

**Figure 2:**
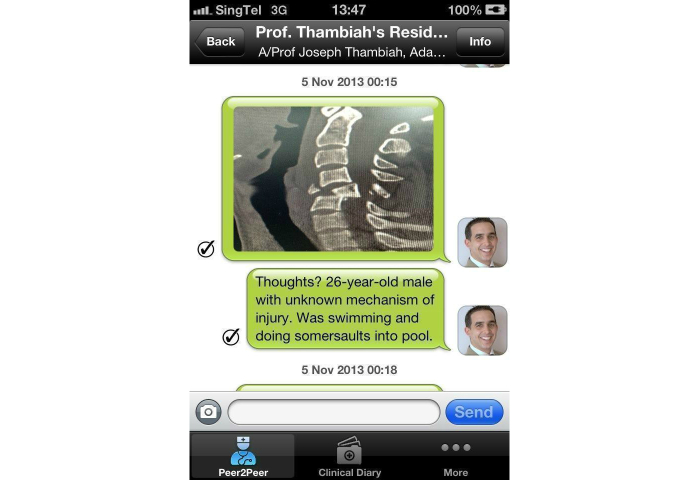
Screenshot of a case discussion including a radiological image.

**Figure 3:**
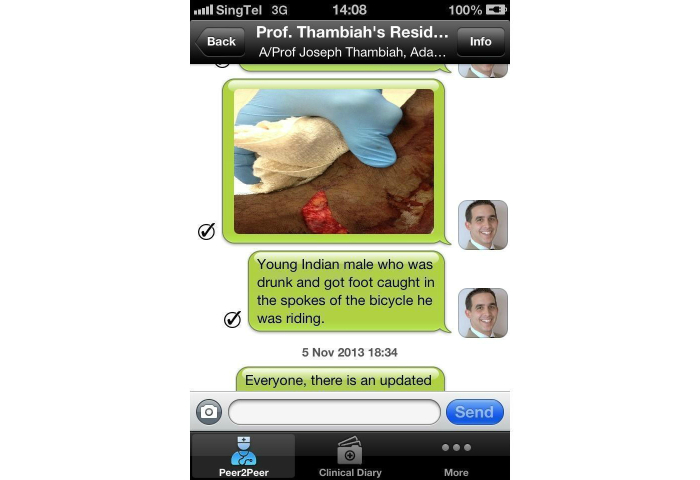
Screenshot of a case discussion including a clinical photograph.

**Figure 4:**
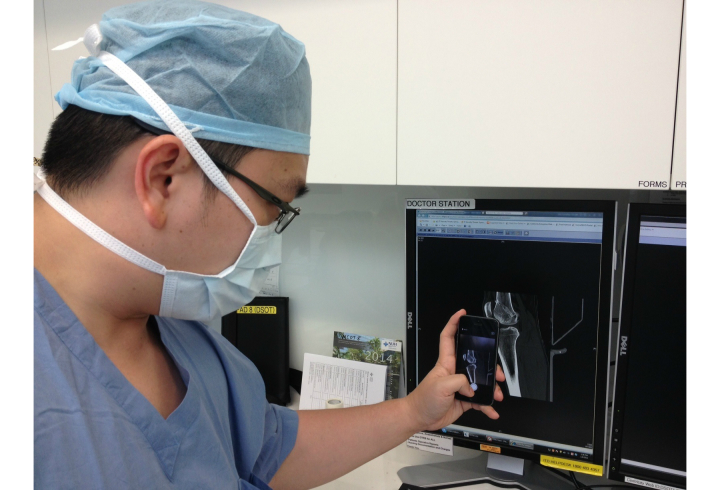
Using an individual mobile phone to take a photo of a radiological image to upload and share through the secure messaging platform.

**Figure 5:**
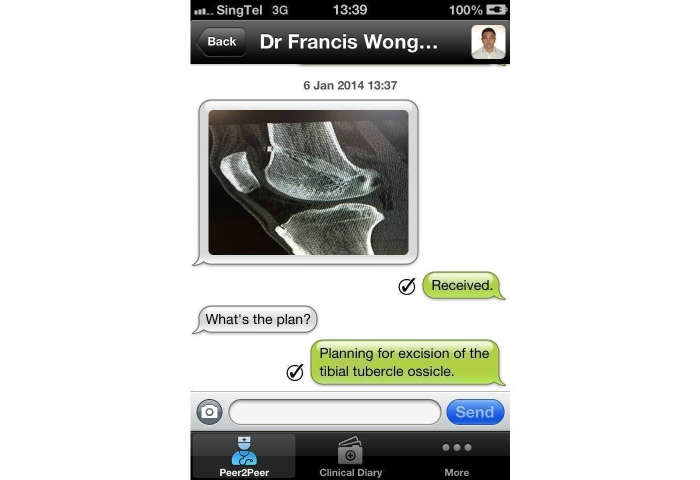
Screenshot of the provision of patient details during a referral.

A prospective study was done to assess the effects of the implementation of an alternative secure messaging system and its effect on staff satisfaction. The orthopedic surgery program director, program coordinator, one trauma consultant, all orthopedic surgical residents, and six non-orthopedic residents at the National University Hospital (NUH) in Singapore were included as participants in the pilot study. All participants were able to understand, speak, and read English. Verbal consent was obtained prior to the study. Our study had a total of 25 staff members with an average age of 32 years (ranging from 25 to 53 years) and comprised of 23 males and 2 females. By using MyDoc as an alternative messaging tool, we commenced communication in the form of personal messages (Figure 1), announcements for residents, case discussions (Figures 2 and 3), as well as providing patient details for referrals. Figure 4 shows a photo of a radiograph being taken to upload on MyDoc for a referral (Figure 5). The feedback from the use of these applications on MyDoc helped determine participants’ satisfaction. At the end of six weeks, the lead author designed a questionnaire based on a previously validated questionnaire [9] and all study participants were asked to complete it.

## Results

All 25 (100%) participants responded to the questionnaire. Almost all (23/25, 92%) participants who filled in the questionnaire agreed (4/25, 16%) or strongly agreed (19/25, 76%) that MyDoc should replace current systems of peer-to-peer communication in the hospital. The remaining two (8%) were unsure.

The majority (22/25, 88%) of participants felt that the quality of images was excellent or very good. Of the remaining 3, 2 (8%) felt they were good and one (4%) felt they were fair. All users (25/25, 100%) felt that the quality of videos and sound was excellent or very good.

Almost all (23/25, 92%) participants also agreed or strongly agreed that they could communicate easily with each other and would feel comfortable doing so through MyDoc on a daily basis. Most of them (20/25, 80%) felt that virtual consults through MyDoc should be made available to inpatients as well as outpatients to potentially lessen clinic loads and provide a secure manner through which patients can communicate with their primary teams at a time and manner convenient to both parties. Of the remaining 5, 3 (12%) were unsure and 2 (8%) disagreed. The majority (22/25, 88%) also agreed or strongly agreed that the potential of telerounding exists and may have advantages, especially when conducted on weekends as a supplement to normal rounds. They also agreed that telerounds could help residents adhere to the number of hours they are allowed to be onsite. Of the remaining 3, 2 (8%) were unsure and one (4%) disagreed.

## Discussion

### Principal Findings

Our pilot study received positive feedback from all staff members with regard to using MyDoc’s secure messaging system. The health insurance portability and accountability act (HIPAA) was enacted by the United States Congress and signed by the President at the time, Bill Clinton in 1996 to give individuals the right to privacy. A summary of the HIPAA can be found on the United States department of health and human services website [10]. Divided into two parts, HIPAA’s first title covers health care access, portability, and renewability. Its second title covers the prevention of health care fraud and abuse, administrative simplification provisions, and medical liability reform. Unsurprisingly, the establishment of national standards for electronic health care transactions and national identifiers for providers, health insurance plans, and employers is a requirement for the second title. While no equivalent act exists in Singapore at present, the closest one, namely the private hospitals and medical clinics act, provides details regarding medical records and the sharing of personal data. It can be found on the Medical Protection Society website [11]. It is thus not surprising that the personal data protection commission (PDPC) of Singapore recently announced the launching of a public consultation exercise to help roll out initiatives to build organizations’ knowledge and capabilities in data protection practices and requirements [12].

While the transmission of personal data in a hospital setting is inevitable, not all methods are secure. This point is reiterated by the American Academy of Orthopedic Surgeons (AAOS), which clearly states that while texting accelerates communication, it puts us at risk and increases liability because it is inherently insecure and incompliant with safety and privacy regulations under the HIPAA [13]. In the month of June 2013 alone, 185,572 messages were sent from the hospital messaging system (HMS) currently in use at the NUH. It should be noted that any service that sends text messages, which are readily accessible to anyone gaining access to the device the messages were sent to, is not completely secure. Considering that the data sent in hospitals is of a sensitive nature, this would be unacceptable should any of these services be used. Although none of the authors of this manuscript condone the words or actions of a medical student who mocked a patient on Twitter earlier this year [14], this example clearly illustrates the dangers of using a social media tool as a platform for transmitting any form of personal data.

In our study, all staff members had very positive reactions towards MyDoc and strongly agreed that this technology be integrated as part of the routine messaging systems currently in use. With traditional teaching claiming that a diagnosis can be made through history alone in 90% of cases and in the remaining 10% with a subsequent physical examination, this emphasizes the pivotal role of communication to the art of medicine. While physical findings are undoubtedly important for making clinical decisions, many decisions are made on gathering this clinical information by conversing with the patient as well as other physicians and nurses. The power of observation and its importance must not be forgotten. For example, during the postoperative ward rounds, wound reviews, dressing changes, and assessment of range of motion require conversational and observational skills. MyDoc includes these in its features. Few clinicians claim that neglecting bedside examination results in medical errors while others suggest that rather than bridging barriers between patients and doctors through bedside interaction, remote presence systems of any form actually increase interactions. Literature however suggests that the use of remote critical care in intensive care units (ICU) managed by internists demonstrated a considerable improvement in measureable patient parameters [15-17].

The provision of specialist care to patients in remote areas who would otherwise have these services unavailable to them only dispels the myth that it increases barriers to appropriate medical care [18]. There are also vast amounts of literature supporting operative telementoring with many studies showing no measurable increase in adverse event rates when patients are operated on by less experienced surgeons supervised by a senior surgeon from a remote location through audiovisual communication [19-24]. In fact, the ethical code and ethical guidelines of the Singapore Medical Council (SMC) clearly states about remote initial consultations in section 4.1.1.2 that,

“No doctor-patient relationship can be established through electronic means and consequently no consultation fee may be received. However, in view of developments in telemedicine and remote-control surgery, it is acceptable for a doctor to manage a patient remotely provided this is in the context of a system of care in which a patient has timely or concurrent access to another doctor who manages him in person. A doctor who provides remote management is responsible for any outcome related to his management”

Regarding remote consultations in continuing care, in section 4.1.1.3, it is mentioned that,

“If a doctor has already established a professional relationship through direct personal contact with a patient, previously made a diagnosis and has commenced treatment, adjusting treatment or providing continued treatment following remote contact with a patient or receipt of electronically transmitted medical data is allowable. If on the other hand it appears from the communication that the patient has developed a new problem or a significant complication, then the doctor shall endeavor to see the patient personally for a further evaluation before offering further treatment.”

The objective of our pilot study was to determine the reaction of our staff over the use of MyDoc in an orthopedic clinical setting in Singapore. Almost all of them found it to be an excellent tool and gave positive feedback. While the introduction and integration of MyDoc may prove to be advantageous in a number of ways, there are various disadvantages and key issues that need to be addressed. For example, the integrity of a secure wireless network and the transmission of sensitive and confidential information is a concern. Ensuring the same is of paramount importance and forms the basis of the implementation of this technology. From a practical point of view, possible loss of the internet connection or network may make the use of the system both impractical and frustrating. Lastly, the speed of message transmission was an issue a number of study participants raised, stating that it is not as fast as other applications (two to three seconds per message or image sent compared to one to two seconds on current messaging applications). However, it must be understood that unlike other systems in use where messages can be read by anyone, forwarded to anyone, remain unencrypted on telecommunication providers’ servers, and most importantly stay forever on the sender’s and receiver’s phones [13], messages on MyDoc are not stored on the phone. The latter, together with the various levels of security, account for the slightly slower transmission speed. These measures, including MyDoc’s assurance of being HIPAA-compliant and having all data stored on a dedicated medical data storage server ensure the security of this messaging platform.

### Potential Uses of MyDoc in Orthopedic Surgery

#### Regional Teleconsults for Specialist Referral

As Singapore is one of the most popular medical hubs in Southeast Asia, the use of virtual consults has the potential to exponentially increase the volume of referrals from around the region. In turn, while the potential increase in patients improves revenue for hospitals and specialists, patients also benefit by the decreased costs they have to face in terms of less travel and accommodation expense as well as the lower doctor’s fees currently in place for a virtual visit.

#### Clinical Outreach in the Setting of Trauma

In tertiary referral centers, the ability to provide greater expertise in the management of trauma patients in centers where specialist expertise is limited has great potential. With the use of MyDoc, inappropriate referrals can also be prevented and appropriate referrals be managed better. Thus the optimal outcome for patients can be achieved by providing expertise at a moment’s notice. This may be most beneficial in the emergency room (ER) setting where often the attending orthopedic surgeon may be scrubbed in the operating room, thereby not being able to attend to the referral at the ER immediately.

#### Supervision in the Operating Room

We have all heard the phrase, “see one, do one, teach one.” Taking this into account, being supervised as opposed to unsupervised is far better for both the trainee and more importantly, the patient. MyDoc has tremendous potential in the operating room as a tool for supervision through its interactive and real time feed. Knowing that they are under the watchful eye of a trainer, reassures the trainees. By the trainer being remotely present, the trainee is forced to think on their feet and be meticulous. While the trainer guides and teaches, the trainee does the procedure themselves. An added benefit includes saving costs by not having the trainer to be on site.

#### Providing Both Patient and Parent Reassurance in Pediatric Orthopedic Patients

In comparison to adult orthopedics where very often we have to deal with just the patient, pediatric orthopedics requires the reassurance of not only the patient who is a child, but also that of the child’s parent or parents. It has been shown that any patient who is discharged from an ER and referred to an orthopedic clinic feels more reassured when they hear the same management plan from the orthopedic surgeon rather than the emergency physician [8]. For this reason, the presence of MyDoc in the emergency department and more so at a pediatric ER, allows the provision of such reassurance. This in turn not only ensures appropriate discharges and referrals but also improves patient satisfaction and lowers costs for unnecessary admissions to the orthopedic surgeon on call.

#### In the Setting of the Outpatient Department

MyDoc may serve a multitude of functions with regard to outpatient clinics. It could help with the concurrent running of two or more clinics, *all* under supervision. Remote clinics could function without added time, travel, and cost constraints. A wound dressing clinic could accept patients without having to wait for the physical presence of a surgeon; as the surgeon could examine the wounds of patients using MyDoc even while running his or her trauma or elective clinic. Furthermore, taking a high-resolution macro photo without running the risk of contaminating the wound allows accurate documentation of what the wound looks like and makes it possible for a different surgeon to objectively assess the healing or worsening of a wound over time. It is noted however, that optimal environmental conditions such as the light intensity would be required and that training on image capturing standards would also need to be done. MyDoc also facilitates the sharing of radiographs between members of an orthopedic team. This however, would also require further validation with users having to be aware of the risks and limitations of such images. While obvious fractures may allow adequate discussion on management, more subtle ones may not.

#### Weekend Ward Rounds

The current economic climate dictates cost savings to be the priority of hospitals. Hence, the necessity for the physical presence of a doctor to perform ward rounds may be substituted by conducting a ward round through the use of virtual consults. This would be applicable only when decisions regarding discharge or necessary management are based more on observational findings rather than the need for clinical examination.

### Conclusions

In early 2013, an article stated that telehealth is expected to grow six fold by 2017 [25]. Subsequently, the fact that telehealth may even be a remedy for chronic hospital readmissions [26] and the reasons why virtual doctor visits are better than in-person ones [27] have been justified. Our pilot study had excellent results in terms of acceptance and satisfaction by all staff members with regard to using MyDoc as a secure messaging system. The integration of the application in an orthopedic clinical setting to provide a variety of other functions as discussed in this paper has an exciting and limitless potential. Downsizing operational costs, despite being beneficial is also needed in order to comply with the Accreditation Council for Graduate Medical Education (ACGME) standards (Singapore has adopted the American system of training) and the recently introduced personal data protection act (expected to be adopted before July 2014). With MyDoc being fully functional on all iPhones already and on the majority of Android devices by end of April 2014 (according to the developers), it is one of the first secure messaging platforms founded and available in Southeast Asia designed explicitly for clinical use. Built by doctors and for doctors, the secure messaging platform MyDoc ticks all the boxes and is something all hospitals and healthcare personnel should consider promoting.

## Abbreviations

ACGME: Accreditation Council for Graduate Medical Education
AAOS: American Academy of Orthopedic Surgeons
HIPPA: health insurance portability and accountability act
PDPC: personal data protection commission
SMC: Singapore Medical Council
